# Kruppel-like factor 15 induces the development of mature hepatocyte-like cells from hepatoblasts

**DOI:** 10.1038/s41598-021-97937-6

**Published:** 2021-09-17

**Authors:** Kazuya Anzai, Kota Tsuruya, Kinuyo Ida, Tatehiro Kagawa, Yutaka Inagaki, Akihide Kamiya

**Affiliations:** 1 Department of Molecular Life Sciences, Tokai University School of Medicine, 143 Shimokasuya, Isehara, Kanagawa 259-1193 Japan; 2 Division of Gastroenterology and Hepatology, Department of Internal Medicine, Tokai University School of Medicine, 143 Shimokasuya, Isehara, Kanagawa 259-1193 Japan; 3 Center for Matrix Biology and Medicine, Graduate School of Medicine, Tokai University, 143 Shimokasuya, Isehara, Kanagawa 259-1193 Japan; 4 Department of Innovative Medical Science, Tokai University School of Medicine, 143 Shimokasuya, Isehara, Kanagawa 259-1193 Japan

**Keywords:** Hepatology, Liver

## Abstract

The liver is an important metabolic organ that controls homeostasis in the body. Moreover, it functions as a hematopoietic organ, while its metabolic function is low during development. Hepatocytes, which are parenchymal cells of the liver, acquire various metabolic functions by the maturation of hepatic progenitor cells during the fetal period; however, this molecular mechanism is still unclear. In this study, Kruppel-like factor 15 (KLF15) was identified as a new regulator of hepatic maturation through a comprehensive analysis of the expression of transcriptional regulators in mouse fetal and adult hepatocytes. KLF15 is a transcription factor whose expression in the liver increases from the embryonic stage throughout the developmental process. KLF15 induced the overexpression of liver function genes in mouse embryonic hepatocytes. Furthermore, we found that the expression of KLF15 could also induce the expression of liver function genes in hepatoblasts derived from human induced pluripotent stem cells (iPSCs). Moreover, KLF15 increased the promoter activity of tyrosine aminotransferase, a liver function gene. KLF15 also suppressed the proliferation of hepatoblasts. These results suggest that KLF15 induces hepatic maturation through the transcriptional activation of target genes and cell cycle control.

## Introduction

The liver is the largest organ in the body that plays an important role in maintaining homeostasis. Owing to its high regenerative ability, when the liver is damaged by some drugs and alcohol, hepatocytes start to proliferate, and the size and functions of the original organ are restored. During the developmental process, the early fetal liver generated from the foregut endoderm has almost no metabolic function and functions as a hematopoietic organ. In the late-fetal stage, blood cells migrate to the bone marrow and spleen, which are the sites of adult hematopoiesis^1^. In contrast, late-fetal hepatocytes mature and acquire the expression of various metabolic enzymes necessary for the function of the adult liver. The expression of liver function genes was induced by the action of oncostatin M (OSM) and the extracellular matrix on hepatic progenitor cells derived from mouse fetal liver^2,3^. OSM is important for liver maturation during the induction of mature hepatocytes from human induced pluripotent stem cells (iPSCs)^4^. In contrast, mature hepatocyte-like cells differentiated from primary hepatic progenitor cells and PSCs in vitro have lower expression of various liver function genes than primary cultured hepatocytes from adult livers. Therefore, the in vitro system for inducing hepatocyte differentiation by the addition of humoral factors is insufficient to induce differentiation into mature liver cells.

In the embryonic development process, the stimulation of several humoral factors can induce the expression of hepatic function-regulating transcription factors in hepatic progenitor cells for hepatic differentiation. Recently, direct reprogramming techniques have enabled the induction of hepatocytes from other cell lineages such as fibroblasts^5,6^. The expression of hepatocyte differentiation factors, such as Hepatocyte nuclear factor (HNF) 4α, FOXA1, FOXA2, HNF1α, and GATA4, is important for hepatocyte lineage specification. In particular, HNF4α is important for the basic functions of hepatocytes and is involved in the formation of cell adhesion structures in hepatic epithelial cells and the regulation of the expression of central enzymes of drug metabolism, such as CYP3A^7^. In contrast, mice deficient in HNF4α in the adult liver are viable, and liver function in HNF4α knockout mice is only partially decreased^8^. Therefore, liver function is regulated by a network of multiple transcription factors. For example, we have previously found that overexpression of the transcription factor Mist1^9^, which is involved in the development of the pancreas, improves liver functions, such as drug metabolism, in mouse fetal liver progenitor cells^10^. Thus, these transcription factors may enhance the function of hepatocytes derived from PSCs. However, the mechanism by which these transcription factors induce hepatocyte differentiation is unclear.

In this study, we considered a group of transcriptional regulators, whose expression changes during liver development, as candidate genes involved in liver function control and conducted a comprehensive screening. As a result, the expression of liver function genes in mouse fetal liver- and human iPSC-derived hepatoblasts can be induced by the overexpression of Kruppel-like factor 15 (KLF15), which is one of the Kruppel-like transcription factors. KLF15 important for the functions of the kidney and heart^11,12^. In addition, KLF15 is involved in drug metabolism in the liver^13^. The expression of KLF15 is induced during the liver maturation process, while the suppression of KLF15 expression by small interfering RNA (siRNA) downregulated the expression of hepatic maturation marker gene. KLF15 also regulates cell proliferation and the expression of cyclin inhibitor p57 in human iPSC-derived hepatoblasts. Based on the above results, we identified KLF15 as a novel factor involved in the regulation of hepatic progenitor cell maturation in this study. In the future, KLF15 can be applied for the functionalization of human PSC-derived hepatocytes.

## Results

### Changes in expression of transcription-related genes during fetal liver development

**Figure 1:**
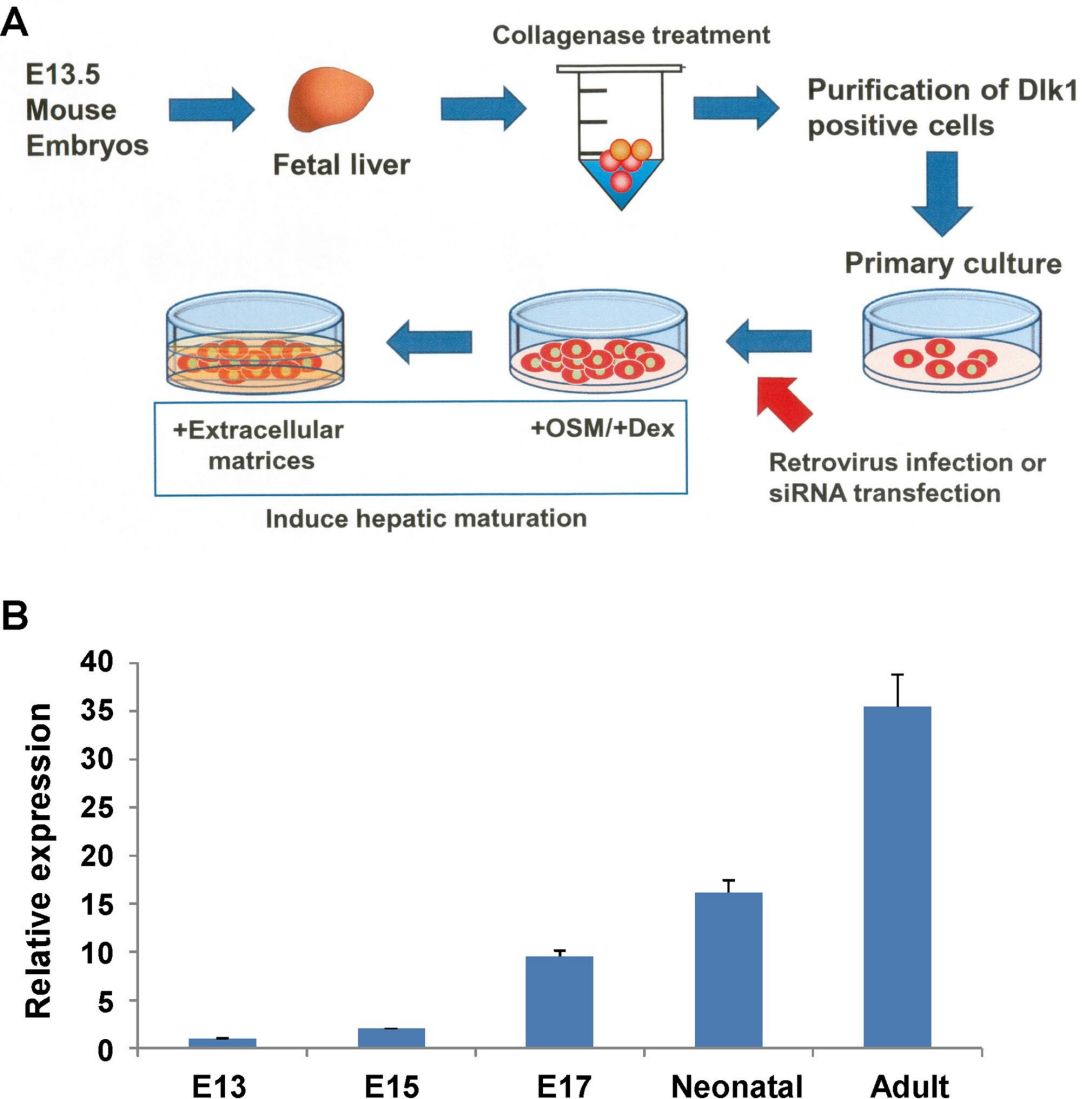
Screening of transcriptional factors that regulate mature liver function. (**A**) The schema of screening system for liver functional factors. Dlk1-positive primary hepatoblasts were purified and cultured on gelatin-coated dishes. Transcription factors were overexpressed using the retroviral vectors. After induction of hepatic maturation by oncostatin M (OSM) and Matrigel, the expression of *Tat*, a liver function gene, was evaluated by quantitative reverse transcription-polymerase chain reaction (RT-PCR). (**B**) The expression of Kruppel-like factor 15 (*Klf15*) in fetal and adult whole livers was analyzed using quantitative RT-PCR. Total RNA was purified from samples derived from embryonic day 13, 15, 17 (E13, E15, E17), neonatal, and 8-week-old adult male livers. Hprt was used as an internal control. Expression of *Klf15* in E13 mouse livers was set to 1.0. Results are represented as the mean ± standard deviation (SD) (n = 4). *P < 0.05.

Hepatoblasts present in the fetal liver primordia differentiate and mature into hepatocytes, which are the major cells responsible for liver function. During this process, hepatocytes acquire the ability to express various metabolic enzymes and liver functional proteins, but the detailed intracellular molecular mechanisms remain unclear. Therefore, we hypothesized that factors whose expression changes during liver development are important for liver differentiation and maturation. Dlk1^+^ hepatoblasts and mature hepatocytes were isolated from the E13 liver and adult liver, respectively, and comprehensive expression analysis was performed by microarray^14^. In this study, multiple nuclear factors with high expression in hepatic progenitor cells and hepatocytes were selected as candidate genes regulating liver function for subsequent analyses (Supplementary Fig. 1). These candidate genes were transferred into mouse fetal liver progenitor cells using a retrovirus, and the expression of tyrosine aminotrannsferase (*Tat*), which is a liver function gene whose expression is increased after birth, was measured (Fig. 1A). Forced expression of KLF15 strongly induced *Tat* expression (Supplementary Fig. 2). Although KLF15 is rarely expressed in the fetal liver, its expression increases as liver development progresses. KLF15 is highly expressed in the adult liver (Fig. 1B). KLF15 is important for the regulation of gluconeogenesis in the liver and skeletal muscles^15^. A previous study using a mouse model with a deletion of the *Klf15* gene (*Klf15* knockout) revealed cardiac hypertrophy characterized by increased heart weight^16^. The response of *Klf15* knockout mice to high-fat feeding revealed that KLF15 was important for endoplasmic reticulum stress and insulin resistance^17^. Adipose-specific *Klf15* knockout mice showed that adipocyte expression of Klf15 was important for adipose triglyceride synthesis and inhibited lipolytic action^18^. However, it is still unknown whether KLF15 is involved in liver development and differentiation. These results suggest that KLF15 may be involved in the development and maturation of fetal liver progenitor cells.

### KLF15 induced maturation of fetal hepatoblasts derived from mouse embryonic livers

**Figure 2:**
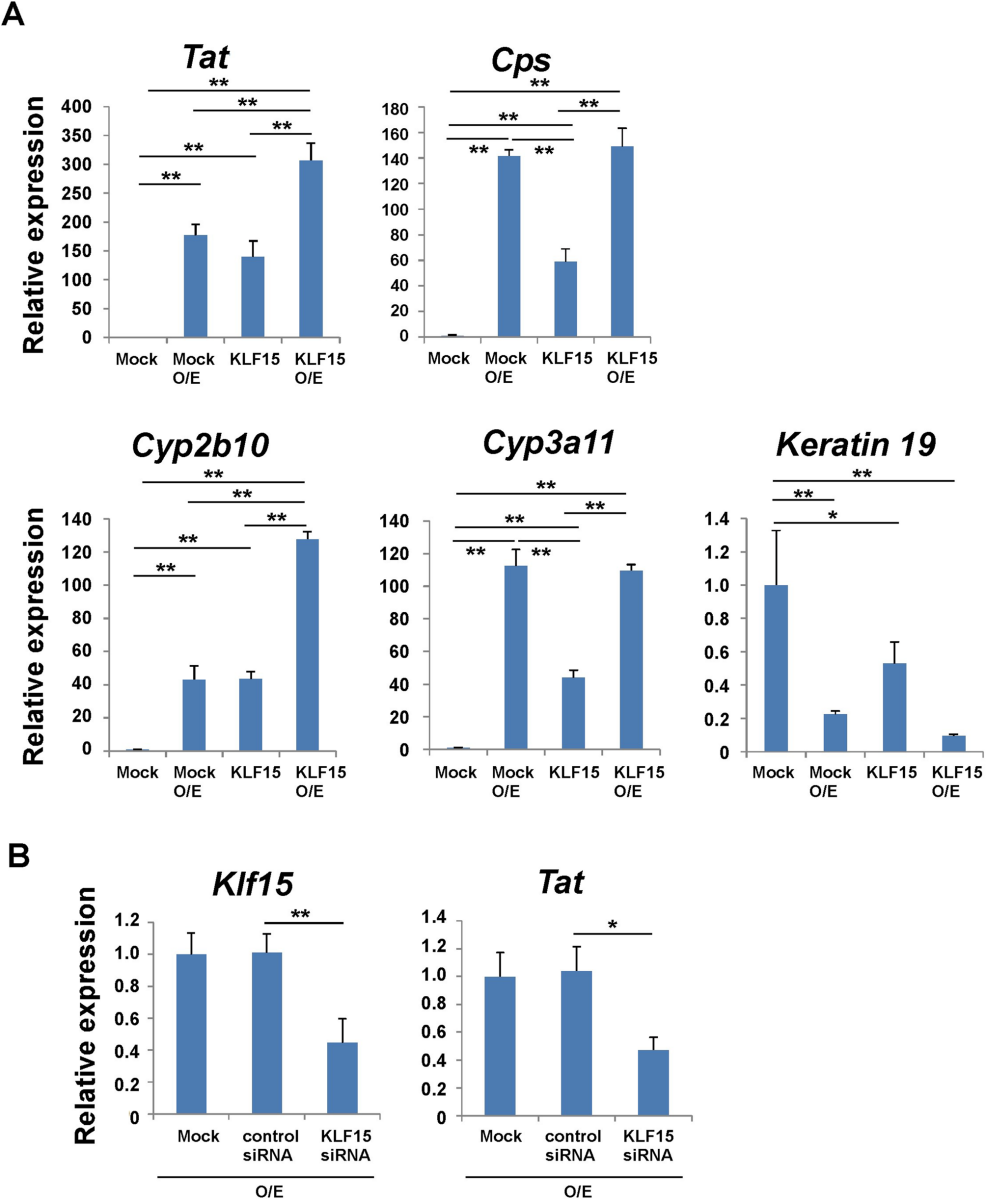
The expression of liver functional genes was regulated by KLF15 in mouse primary hepatoblasts. (**A**) Overexpression of KLF15 in fetal hepatoblasts. As shown in Fig. 1A, after culturing, the expression of *Tat*, *Cps*, *Cyp2b10*, *Cyp3a11* (hepatic genes), and keratin 19 (cholangiocytic gene) were analyzed using quantitative RT-PCR. The expression of genes in cells infected with the mock vector was set to 1.0. (**B**) Knockdown of endogenous KLF15 in hepatoblasts maturation. Hepatoblasts were transfected with *Klf15* siRNA by lipofection, and the expression of *Klf15* and *Tat* was analyzed using quantitative RT-PCR. The expression of genes in cells infected with the control siRNA was set to 1.0. Results are represented as the mean expression ± SD (n = 3). *P < 0.05, **P < 0.01. O/E; OSM+extracellular matrix (Matrigel).

Mouse fetal liver hepatoblasts were isolated, purified with DLK1 antibody, and KLF15 was transduced using a retrovirus vector. Hepatic maturation was induced by stimulation with liver maturation factors (OSM and the extracellular matrix)^2,3^. The expression of mature hepatocyte markers, such as those of amino acid metabolism (*Tat*), urea synthesis (carbamoyl phosphate synthetase 1, *Cps1*), drug metabolism (cytochrome P450, *Cyp*), or the cholangiocytic cell marker (Keratin 19), was analyzed by quantitative reverse transcription-polymerase chain reaction (RT-PCR) (Fig. 2A). The combination of KLF15 overexpression and liver maturation factors significantly induced the expression of *Tat* and *Cyp2b10*. We recently reported that mouse fetal hepatoblasts began to differentiate into cholangiocytic cells in vitro culture without the addition of the liver maturation factors OSM and extracellular matrices^19^. In contrast, gene transfer of KLF15 increases the expression of mature hepatocyte markers even without the addition of these liver maturation factors. Furthermore, KLF15 suppressed the expression of Keratin 19, suggesting that KLF15 promoted differentiation into hepatocytes and suppressed cholangiocytic differentiation.

Next, when the expression of *Klf15* was suppressed by siRNA transfection, expression of the hepatocyte maturation marker *Tat* was analyzed (Fig. 2B). As a result, it was found that the expression of *Tat* was suppressed as the expression of KLF15 decreased. In addition, the expression of liver-enriched factors was analyzed in both *Klf15*-overexpressing and -knockdown cultures (Supplementary Fig. 3 and 4). Several transcriptional factors were expressed in E13 hepatoblast culture. In particular, HNF4α expression was significantly induced by the hepatic maturation factor (OSM and extracellular matrices) with and without *Klf15* overexpression. However, both *Klf15* overexpression and knockdown did not alter the expression of these transcriptional factors. Thus, it is suggested that KLF15 induces hepatic maturation independently of the induction of these factors.

**Figure 3:**
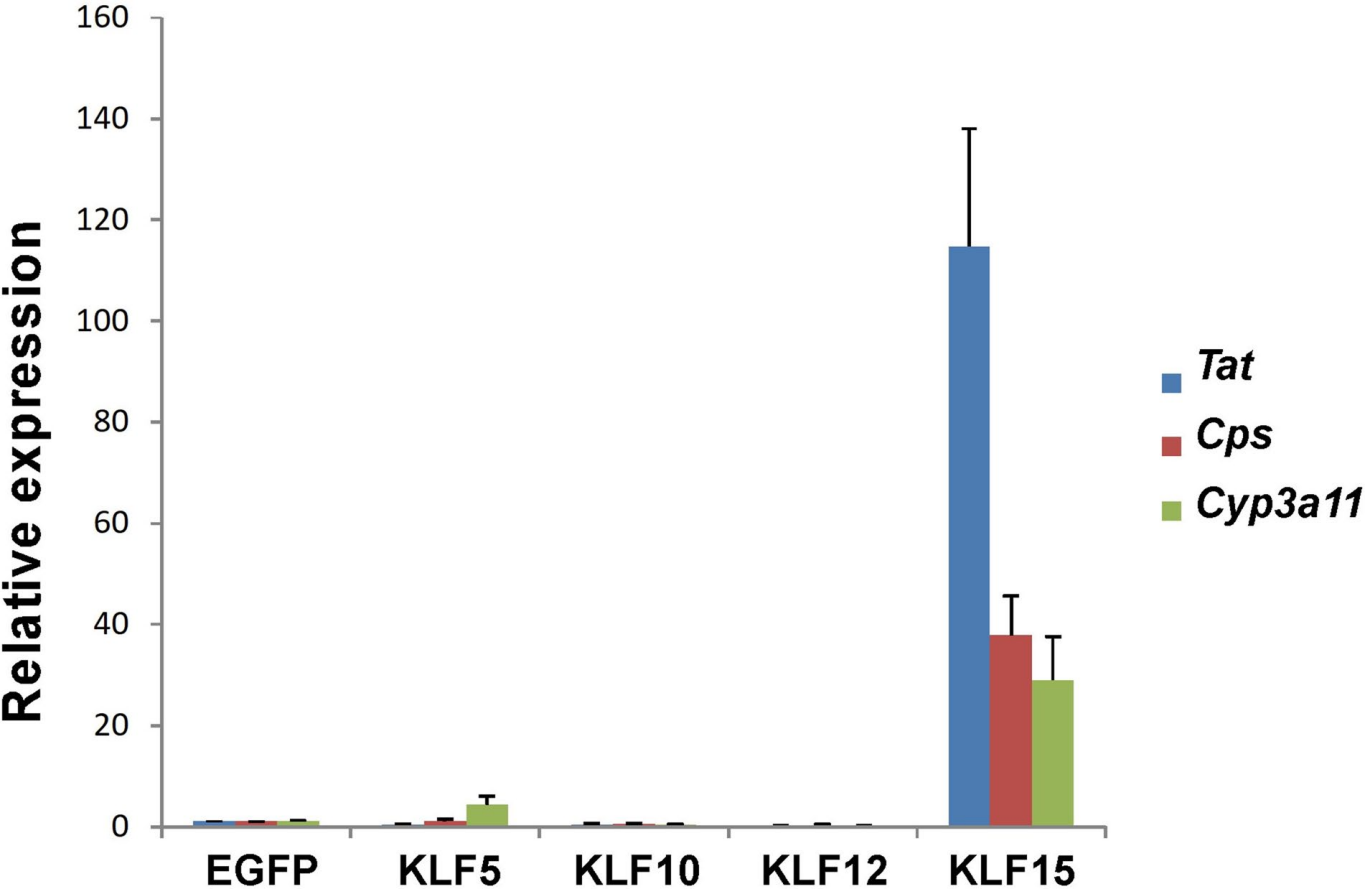
The expression of liver functional genes was regulated by KLF15 but not by other KLF family transcription factors. After overexpressing KLF15 and other KLF family genes in fetal hepatoblasts, the expression of liver functional genes was analyzed using quantitative RT-PCR. Expression of genes in cells infected with the mock vector was set to 1.0. Results are represented as the mean expression ± SD (n = 2).

KLF is a family of transcription factors with a zinc-finger DNA-binding region at the C-terminus. For example, both KLF5 and KLF15 have been reported to be important for adipocyte function and differentiation^18,20^. Therefore, we analyzed whether other factors in the KLF family could promote liver maturation as KLF15 did (Fig. 3). KLF15 could effectively promote hepatic maturation, whereas other KLF family transcription factors, KLF 5, 10, and 12, showed almost no such activity. In addition, the expression of KLF transcription factors was analyzed in E13 hepatoblast cultures. Expression of *Klf15* and other *Klf* family genes (*Klf5*, *10*, and *12*) was detected in the hepatoblast culture (Supplementary Fig. 5). *Klf15* expression was upregulated (approximately five-fold compared to E13 primary hepatoblasts) during seven days of culture with hepatic maturation factors (OSM and the extracellular matrix). In contrast, other KLF family genes were barely upregulated compared to primary E13 hepatoblasts. From the above results, we identified KLF15 as an important factor in the maturation of hepatic progenitor cells.

### KLF15 induced maturation of hepatoblasts derived from human iPSCs

**Figure 4:**
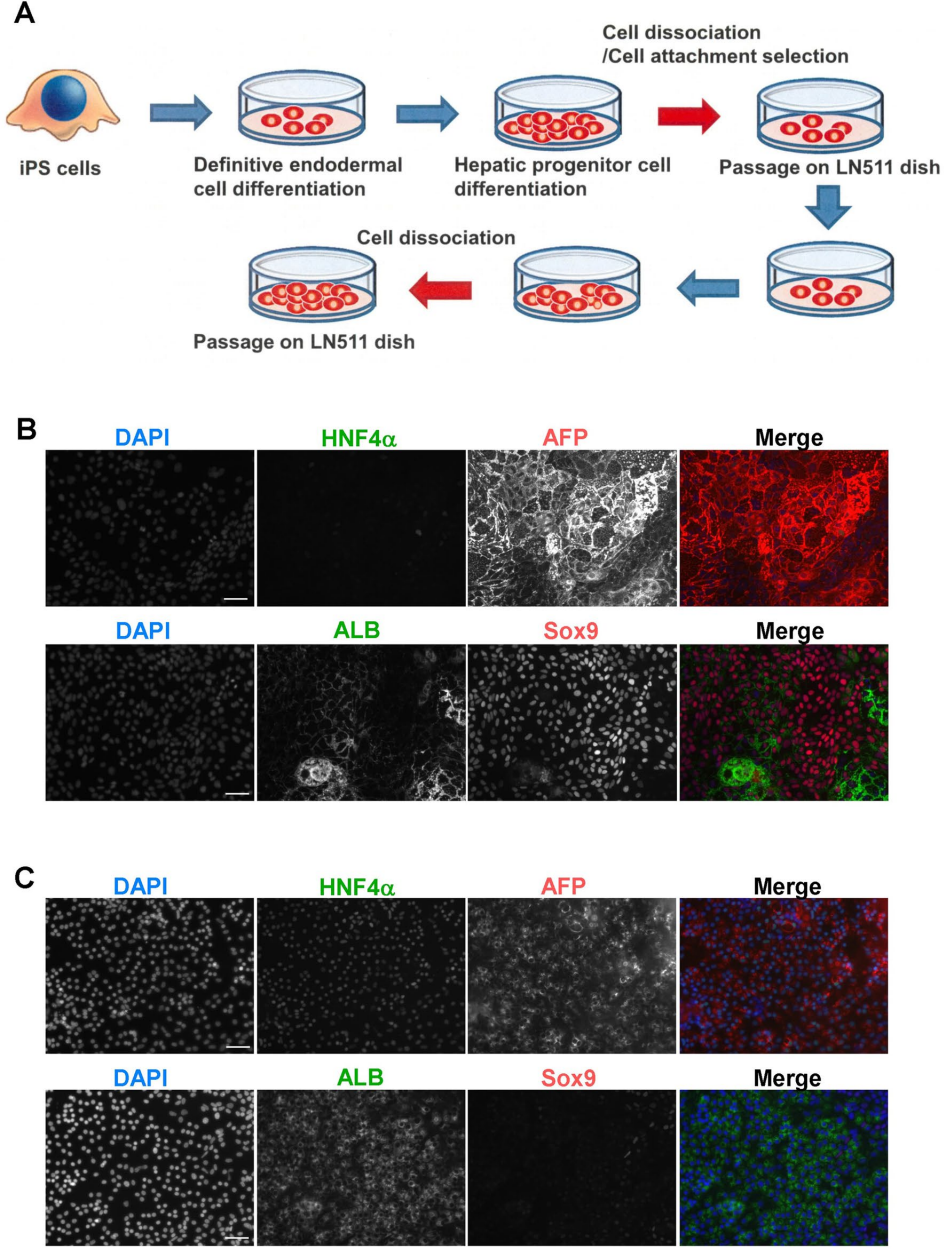
Establishment of human induced pluripotent stem cell (iPSC)-derived hepatoblasts. (**A**) The schema of the culture system of hepatoblasts derived from human iPSCs. Differentiation of human iPSCs into definitive endodermal and hepatic progenitor cells was induced under the suitable culture conditions. These cells were passaged several times on LN511-coated dishes. Expanded cells were used as human hepatoblasts. (**B**,**C**). Expression of progenitor and hepatocyte marker proteins of human hepatoblasts (**B**) and differentiated hepatocytes (**C**) derived from iPSCs. Passaged hepatoblasts were cultured and fixed using 4% paraformaldehyde. These cells were stained with hepatocyte marker proteins (ALB and HNF4α) and hepatic progenitor marker proteins (AFP and SOX9). Scale bar, 50 μm.

**Figure 5:**
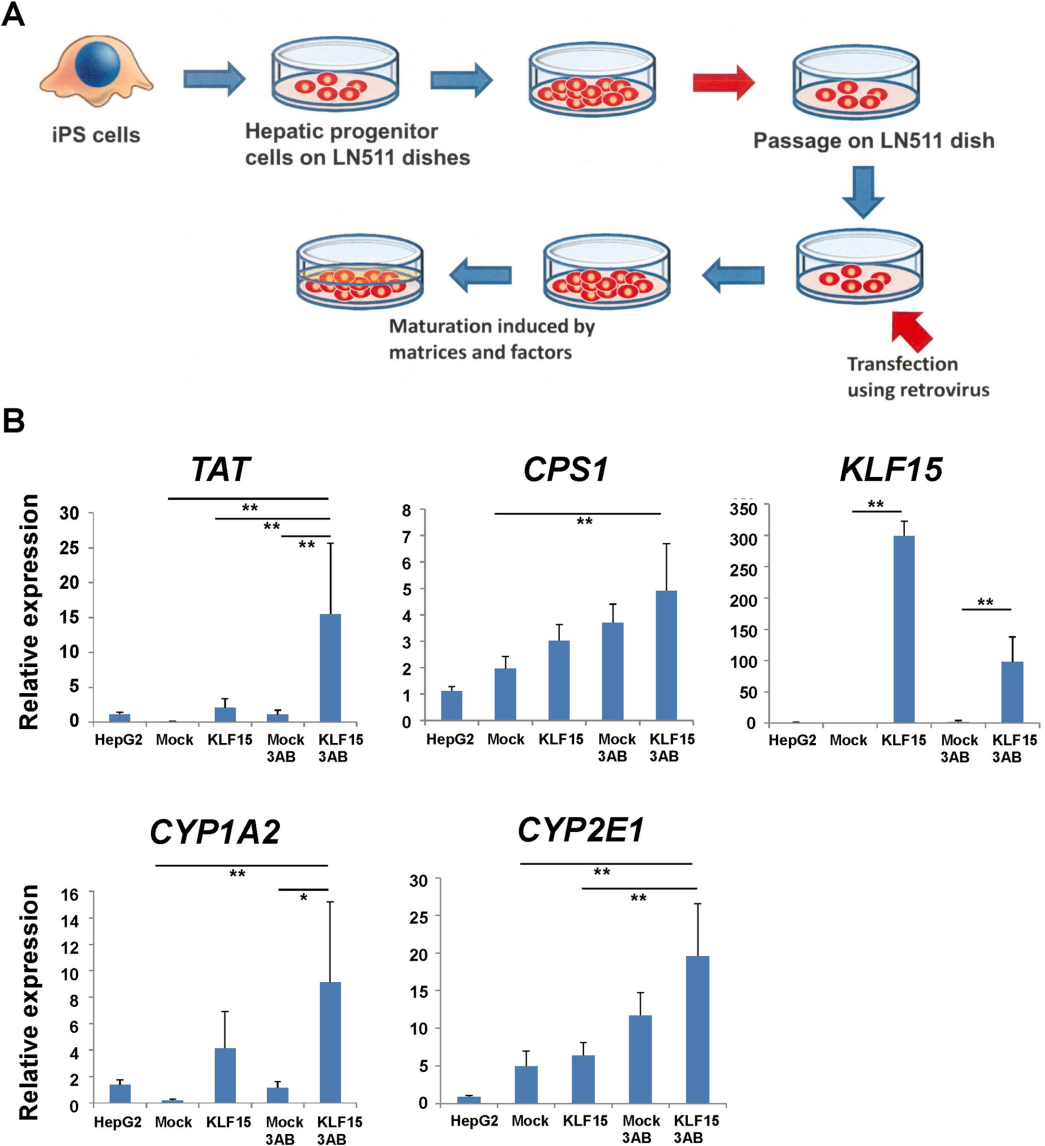
Induction of hepatic maturation by KLF15 in human iPSC-derived hepatoblasts. (**A**) The schema of the gene transduction system of hepatoblasts derived from human iPSCs. Human hepatoblasts derived from iPSCs were cultured on LN511-coated dishes and infected with retroviral vectors to induce gene expression. After infection, hepatic maturation was induced under suitable culture conditions. (**B**) Overexpression of KLF15 in human hepatoblasts. As shown in (**A**), after culturing, the expression of *TAT*, *CPS1*, *CYP1A2*, *CYP2E1*, and *KLF15* were analyzed using quantitative RT-PCR. Gene expression in cells infected with the mock vector was set to 1.0. Results are represented as the mean expression ± SD (n = 3). *P < 0.05, **P < 0.01.

Human iPSCs are considered an important resource in regenerative medicine because of their high proliferative potential and pluripotency. Various differentiation-inducing methods for iPSC-derived hepatocytes have also been reported^21^. In this study, we induced the differentiation of hepatoblasts from human iPSCs and established a system capable of maintaining long-term culture. Human iPSCs were induced by the endodermal progenitor cell and hepatic progenitor differentiation media^22^. These hepatoblasts were then seeded on an LN511-coated culture dish and cultured for 7 days in a hepatic colony-forming medium. Through several subculturing steps, the obtained human iPSC-derived hepatoblasts were capable of long-term proliferation (Fig. 4A). Under normal subculture conditions, these cells barely expressed HNF4α and albumin (ALB) proteins, which are markers for differentiated hepatocytes (Fig. 4B). In contrast, they strongly expressed the hepatic progenitor cell markers AFP and SOX9. When these cells were cultured in a hepatocyte differentiation medium containing extracellular matrices (3AB medium, as described in the Methods section), the production of HNF4α and ALB was strongly induced, while the level of SOX9 was decreased (Fig. 4C). The above results suggested that the established cells proliferated as progenitor cells and also functioned as mature hepatocytic-like cells under appropriate hepatocyte differentiation culture conditions. Next, KLF15 was overexpressed in human iPSC-derived hepatoblasts, and its effect on hepatocyte differentiation was observed. Hepatoblasts were seeded in LN511 culture dishes, *KLF15* was overexpressed using retrovirus vectors, and hepatic maturation was induced by hepatocyte differentiation medium 3AB (Fig. 5A). As shown in Supplementary Fig. 6, the expression of *ALB* and *HNF4*α mRNA, which are hepatocyte marker genes, was significantly induced upon addition of hepatocyte differentiation medium (3AB) both with and without *KLF15* overexpression conditions. The expression of the mature hepatocyte markers *TAT*, *CPS1*, *CYP1A2,* and *CYP2E1* was also analyzed in human iPSC-derived hepatoblast cultures. A higher induction of hepatocyte marker expression was observed by combining the overexpression of *KLF15* overexpression and hepatic differentiation medium (KLF15 + 3AB, Fig. 5B). In particular, the expression of *TAT* and *CYP1A2* was significantly increased by the addition of both *KLF15* and 3AB medium compared to 3AB medium alone. Thus, these genes may be more efficiently regulated by KLF15. These results suggest that KLF15 plays an important role in the induction of human hepatocyte differentiation.

### Mechanisms regulating hepatic maturation of human iPSC-derived hepatoblasts through KLF15

**Figure 6:**
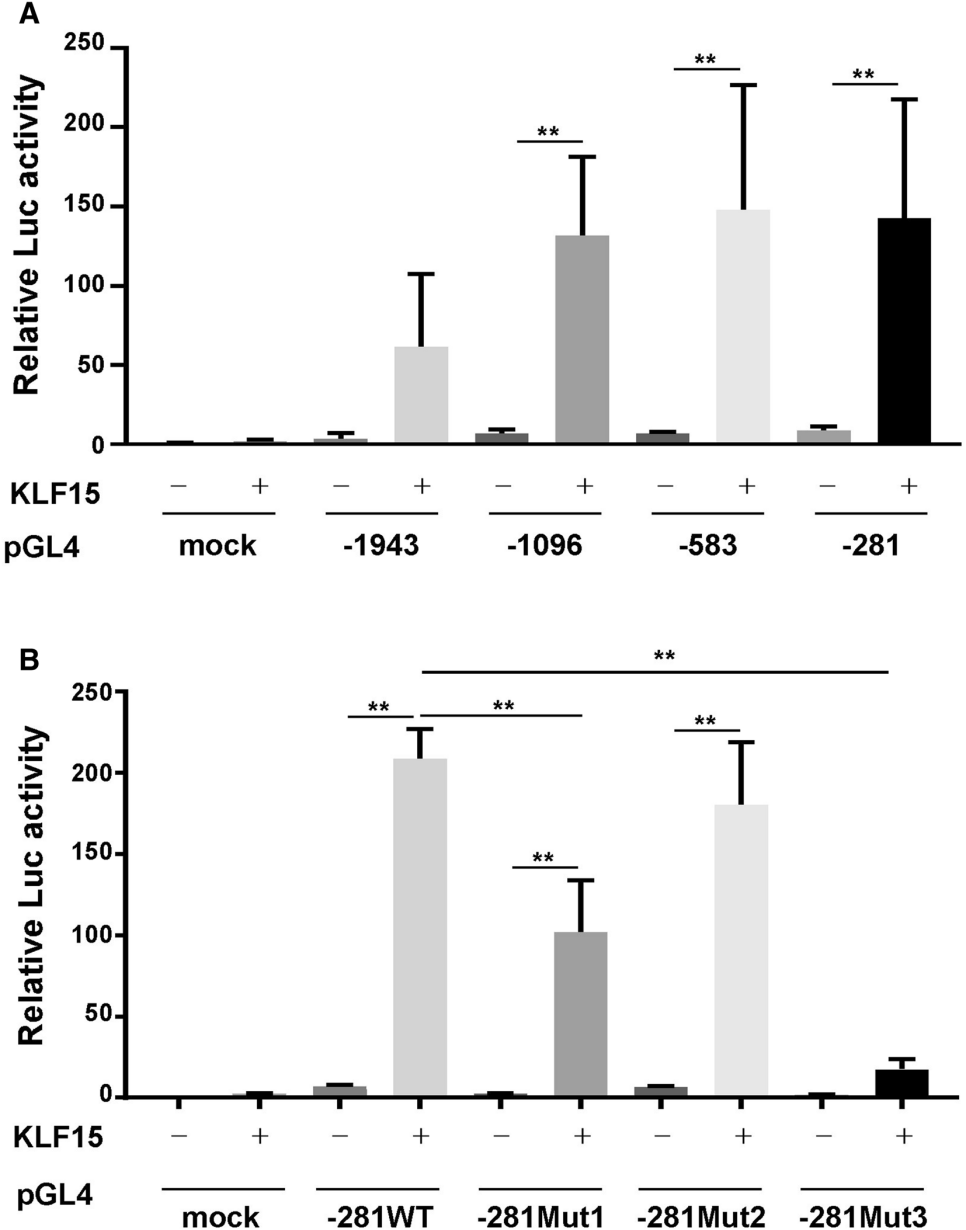
Promoter region of the human *TAT* gene regulated by KLF-binding region. (**A**) The promoter activity of *TAT* was analyzed by luciferase assay. Several truncated *TAT* promoter and *KLF15*-overexpressing vectors were cotransfected into HepG2 hepatoma cells. Results are presented as the mean activity ± SD (n = 4; vector of -1943, n = 3). (**B**) The promoter activity of the wild-type (WT) and mutated -281 TAT promoter regions (Mut1-3) was analyzed by luciferase assay. Several mutated *TAT* promoter and *KLF15*-overexpressing vectors were cotransfected into HepG2 hepatoma cells. Results are presented as the mean activity ± SD (n = 3). **P < 0.01.

We analyzed the molecular mechanism by which KLF15 induced the maturation of human iPSC-derived hepatoblasts. Through analysis of the upstream region of the liver differentiation marker gene *TAT*, whose expression was induced by KLF15, we found putative KLF consensus sequences near the transcription start site of *TAT* (Supplementary Fig. 7A, the green oligonucleotides). Therefore, the promoter region of *TAT* was cloned, and its transcriptional activity was analyzed by the luciferase assay (Fig. 6A). The transcriptional activity was increased by overexpression of KLF15 in the promoter region (− 281 bp upstream) of the transcription initiation site, which has the predicted binding sequences of KLF15. Next, we prepared mutated promoter vectors that replaced these putative consensus sequences (Supplementary Fig. 7B). The luciferase activity induced by KLF15 was slightly decreased by the mutation of binding site 1. In addition, the mutation of binding site 3 suppressed the KLF15-derived induction of the − 281 *TAT* promoter (Fig. 6B). These results suggest that *TAT* expression is directly induced by KLF15 through the proximal promoter region.

**Figure 7:**
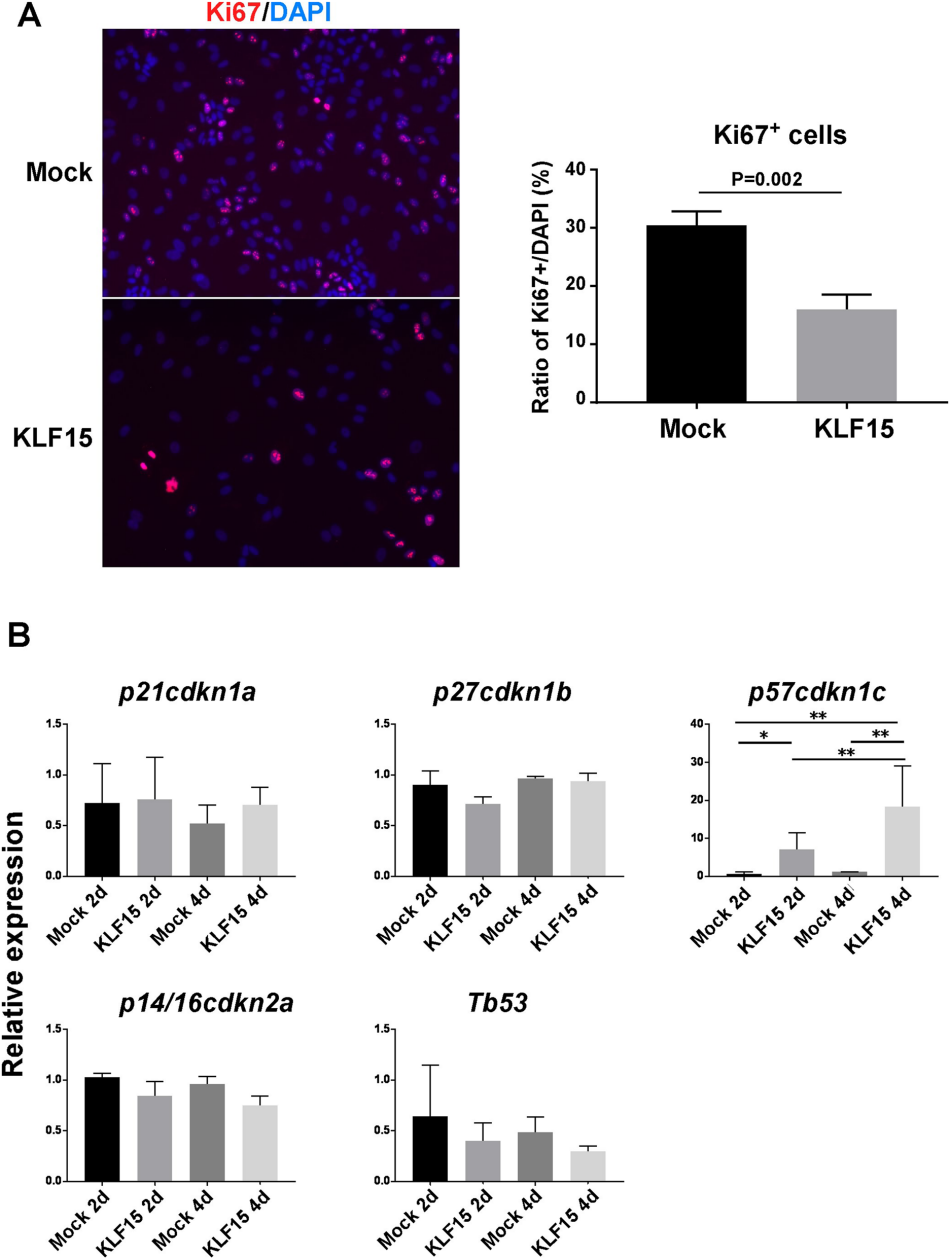
Regulation of cell proliferation and the cell cycle by KLF15 in human hepatoblasts. (**A**) Human hepatoblasts derived from iPSCs were cultured and infected with control or KLF15-overexpressing retrovirus vector. After 4 days of culture, DAPI- and Ki-67-positive cells were counted. Results are represented as the mean ± SD (n = 3). (**B**) Expression of cell cycle-related genes in human hepatoblasts. Human hepatoblasts derived from iPSCs were cultured and infected with mock or KLF15-overexpressing retrovirus vector. After 2 and 4 days of culture, RNA was extracted, and gene expression was analyzed by quantitative RT-PCR. The expression of genes in cells infected with the mock vector (2 days of culture) was set to 1.0. Results are represented as the mean expression ± SD (n = 3). *P < 0.05, **P < 0.01.

KLF15 also induces the expression of other liver function genes. It is suggested that another mechanism is related to the KLF15-induced hepatic differentiation. The cell proliferation ability of KLF15-overexpressing hepatoblasts was quantified by the expression of Ki67 (Fig. 7A). KLF15 suppressed the proliferation of human iPSC-derived hepatoblasts. We analyzed the changes in the expression of Cdk inhibitors that control the cell cycle. Expression of p57cdkn1c was increased in the KLF15-overexpressing hepatoblasts (Fig. 7B). While the proliferative capacity of undifferentiated cells is high, their proliferative capacity is often suppressed during cell differentiation and maturation. Therefore, it is possible that suppression of the high proliferative capacity of progenitor cells by KLF15 is an important factor that induces cell differentiation.

## Discussion

In this study, we identified a novel molecular mechanism that induces the maturation of hepatic progenitor cells. The developing liver is characterized by drastic functional changes from a hematopoietic organ to a metabolic organ. Therefore, changes in properties during the differentiation of hepatocytes, which are mainly responsible for liver function, are important. We hypothesized that this process is regulated by various transcriptional regulators whose expression changes during liver development and maturation. During mouse embryonic development, the immature hepatic progenitor cells have a few metabolic functions, but have hematopoietic support, such as cytokine secretion and cell–cell interaction. In contrast, mature hepatocytes express several liver function genes, such as genes related to drug metabolism and amino acid metabolizing enzymes. Therefore, we comprehensively analyzed transcriptional regulators that show differential expression between fetal hepatoblasts and mature hepatocytes and searched for factors that alter the expression of liver function genes. In our previous study, we reported that the transcription factor Mist1, whose expression is temporarily increased during liver development, induces the maturation of mouse hepatic progenitor cells^10^. In this study, approximately 40 transcriptional factors, whose expression changed during liver development, were evaluated for their ability to induce expression of a liver function gene *Tat* in hepatic progenitor cells. Among the factors analyzed in this study, KLF15 was identified as a novel transcription factor regulating liver functional maturation. In our previous studies, we also found that the addition of a humoral factor (differentiation-inducing medium) and the extracellular matrix induced maturation of hepatic progenitor cells^2,3^. In contrast, KLF15 partially induced the expression of hepatic function genes without the involvement of other humoral maturation factors in mouse hepatoblasts culture. Therefore, there may be some mechanisms that control liver maturation downstream of KLF15. In this study, we found that KLF15 is involved in the induction of p57^cdkna1c^ expression and suppression of cell proliferation. Cell proliferation is known to be downregulated during various cell differentiation processes. The decrease in cell proliferation due to KLF15 may be related to its ability to induce hepatic differentiation in this culture system.

KLF15, a transcription factor belonging to the KLF family, which are important for various cell differentiation processes. For example, KLF2 is involved in the reprogramming of somatic cells into pluripotent cells. In particular, KLF15 is known to be involved in adipocyte differentiation and hepatic fat metabolism, similar to KLF5^20^. The overexpression of KLF5 and other KLF family molecules did not promote liver maturation markers, as observed in KLF15. Analysis of the promoter region of *TAT*, a liver maturation marker, revealed that there are several KLF-binding regions, and mutations of these sites significantly suppressed the activation of the *TAT* promoter region induced by KLF15. This suggests that this region is important for the promoter activity. In addition, we analyzed the sequence of the − 1500 bp region upstream of the *CYP1A2* promoter, and several oligonucleotide sequences were identified as binding sites of KLF15 and other KLF families showing particularly high binding scores. These regions may be directly related to the induction of *CYP1A2* expression by KLF15. In addition, regarding the promoter region of *cdkn1c*, there is a highly GC-rich region in the proximal promoter of *cdkn1c*. The conserved binding sequence of KLF15 is also a GC-rich sequence, so it is possible that KLF15 binds to this GC-rich region. How KLF15 regulates *CYP1A2* and *p57cdkn1c* promoter activities should be looked into in future studies.

Overall, KLF15 was identified as a novel regulator that promotes the maturation of hepatoblasts. Hepatocyte progenitor cells and hepatocytes derived from human PSCs are expected to have various uses, such as cell transplantation therapy and drug discovery screening systems. Noteworthily, the sufficient expression of drug-metabolizing enzymes or other liver maturation genes for these applications was not observed in the hepatic differentiation culture system used in our previous study. The screening system shown in this study might be useful to clarify the molecular mechanism involved in liver maturation and identify important transcription factors, which will lead to the identification of more hepatocyte-inducing factors.

## Methods

### Materials

C57BL/6N mice were purchased from Nihon SLC (Shizuoka, Japan). Animal experiments were performed with the approval of the Institutional Animal Care and Use Committee of Tokai University (approval number: #204009), confirming that all experiments were performed in accordance with relevant guidelines and regulations. Dulbecco’s modified Eagle’s medium (DMEM), DMEM/Ham’s F12 medium, penicillin/streptomycin/L-glutamine (100 ×), dexamethasone, nicotinamide, and gelatin from porcine skin were purchased from Sigma-Aldrich (St Louis, MO, USA). Insulin-transferrin-selenium, non-essential amino acids, and HEPES buffer were purchased from Thermo Fisher Scientific (Carlsbad, CA, USA). Fetal bovine serum (FBS) was purchased from Nichirei Biosciences (Tokyo, Japan). Hepatocyte growth factor (HGF) and epidermal growth factor (EGF) were purchased from PeproTech (Rocky Hill, NJ, USA). Y-27632 and A-83-01 were purchased from Wako Pure Chemical Industries (Osaka, Japan). Human iPS cell line ChiPSC18 was purchased from Takara Bio Inc. (Shiga, Japan).

### Isolation of hepatic progenitor cells from mouse fetal livers

Purification and culture of fetal mouse hepatoblasts were performed as previously described^10^. Embryonic day (E) 13 C57BL/6N mouse fetal livers were minced and digested with liver perfusion buffer (0.5 mM EGTA solution) and liver digest medium (0.05% collagenase solution). These cells were incubated at 4 °C for 30 min with biotin-conjugated anti-CD45 and biotin-conjugated anti-Ter119 antibodies (BioLegend, San Diego, CA, USA). Contaminating hematopoietic cells were excluded using DynaMag™ 15 with Dynabeads™ MyOne Streptavidin C1 (Thermo Fisher Scientific). Subsequently, Dlk1^+^ cells were selected and purified using magnetic-activated cell sorting (MACS) technology (Miltenyi Biotec, Bergisch Gladbach, Germany) using an anti-Dlk1 antibody (Preadipocyte factor-1, Medical and Biological Laboratories, Nagoya, Japan). CD45^-^Ter119^-^Dlk1^+^ cells were eluted from the MACS LS column (Miltenyi Biotec) and used as the mouse fetal hepatoblast fraction.

For microarray analyses, minced embryonic liver cells were stained with FITC-conjugated anti-Dlk1, allophycocyanin-conjugated anti-CD133 (eBioscience, San Diego, CA, USA), and PE-cy7 conjugated anti-Ter119, -CD45, and -c-Kit (eBioscience) antibodies at 4 °C for 60 min. After the washing step, cells were analyzed, and Dlk1^+^CD133^+^Ter119^-^CD45^-^c-Kit^-^ cells were sorted by fluorescence-activated cell sorting (FACS) using a FACS Aria I and III (BD Biosciences, San Jose, CA, USA). The antibodies used for cell purification are listed in Supplementary Table 1.

### Purification of adult hepatocytes for microarray analyses

Adult hepatocyte purification was performed as previously described^10^. Briefly, 8-week-old male mice were subjected to a standard two-step collagenase perfusion. The liver was pre-perfused through the portal vein with 0.5 mM EGTA solution and perfused with 0.025% collagenase (Yakult, Tokyo, Japan) solution. Hepatocytes were purified using 50% Percoll™ (GE Healthcare UK Ltd., Little Chalfont, UK) buffer and then centrifuged at 50 × g for 10 min.

### Transcription profile analysis using microarrays

As described previously, purified fetal hepatoblasts and adult hepatocytes were used for the microarray analyses^14^. Total RNA was purified from these cells using the RNeasy Micro Kit (Qiagen, Victoria, Australia), according to the manufacturer’s instructions. Transcription profiles were analyzed using the Agilent Whole Mouse Genome Microarray 4 × 44 K. The original data are available from the Gene Expression Omnibus (accession number GSE56734) ^14^ (Ito et al.). Expression data were analyzed using the Gene Springs. Datasets were normalized, and transcription-related genes with differential expression during in vivo liver development were extracted and represented as a heat map.

### Generation of retrovirus for gene transduction

The retroviral vector pGCDNsam was used for gene transduction into fetal hepatoblasts and human iPSC-derived hepatoblasts^23^. The complementary DNA (cDNA) of transcription factors was subcloned into an upstream sequence of an internal ribosomal entry site (IRES) and enhanced green fluorescent protein in a pGCDNsam vector. Infected cells can be detected using a fluorescent microscope. Retroviruses were generated as previously described^24^. The same titer of viruses was added to the cultured cells.

### Culture and gene transduction of mouse fetal hepatoblasts

Approximately 1 × 10^5^ Dlk1^+^ hepatoblasts per well were cultured on 0.1% gelatin-coated 24-well plates in hepatocyte culture media: DMEM supplemented with 10% FBS, 1 × minimal essential medium (MEM) non-essential amino acid solution, insulin-transferrin-selenium, 10^–7^ M dexamethasone, and penicillin–streptomycin-glutamate. For hepatic maturation, cells were cultured with OSM (R&D Systems, Inc., Minneapolis, MN, USA) and Matrigel (BD Biosciences), as previously described^3^. For the Matrigel gel overlay, the culture medium was removed, and Matrigel diluted in ice-cold hepatocyte culture media with OSM at a volume ratio of 1:5 (Matrigel/Medium) was added to the culture dishes.

For gene overexpression, pGCDN retrovirus infection was performed after plating the fetal hepatoblasts. For the gene knockdown assay, siRNA transfection was performed using X-treme Gene siRNA Transfection Reagent (Roche Diagnostics) according to the manufacturer’s protocol. siRNAs were purchased from Dharmacon (Lafayette, CO, USA). The cells were harvested at the indicated times, depending on the analysis. Total RNA was extracted using RNAiso Plus (Takara Bio Inc.).

### Isolation of fetal, neonatal, and adult livers for expression analysis

Embryonic day (E) 13, 15, and 17 as well as neonatal livers were excised under a microscope and stored in RNAlater (Thermo Fisher Scientific). Adult livers were excised after bleeding out the mice and stored in RNAlater. Total RNA was extracted using RNAiso Plus.

### Detection of mRNA by quantitative RT-PCR

First-strand cDNA for quantitative RT-PCR was synthesized using the ReverTra Ace qPCR RT Master Mix with gDNA Remover (TOYOBO, Osaka, Japan) or the High-Capacity cDNA Reverse Transcription Kit (Thermo Fisher Scientific). The expression of the target genes was normalized to that of hypoxanthine–guanine phosphoribosyl transferase (*Hprt*) or TATA-binding protein (*TBP*). Quantitative analysis of target mRNA was performed using the Universal Probe Library System (Roche Diagnostics, Basel, Switzerland). The primers and probes used for quantitative RT-PCR are listed in Supplementary Table 2.

### Differentiation of human iPSCs towards hepatic lineage cells in vitro

The differentiation protocol for induction of hepatocytes was based on our previous report^22,25^ with some modifications. Feeder-free human iPSC culture was performed using the Cellartis DEF-CS Culture System (Takara Bio Inc.). These iPSCs were passaged every 4 to 7 days to maintain an undifferentiated state. The Cellartis iPS Cell to Hepatocyte Differentiation System (Takara Bio Inc.) was used to differentiate human iPSCs into hepatoblasts-like cells, according to the manufacturer’s protocol.

Hepatoblasts-like induced from human iPSCs were trypsinized using 0.05% trypsin–EDTA (Sigma, St Louis, MO) and cultured on Laminin 5–1-1 fragment (iMatrix-511, Takara Bio Inc.)-coated dishes. Standard culture medium, which is a 1:1 mixture of hepatic colony-forming unit (H-CFU-C) medium and DMEM with 10% FBS and 10^−7^ M dexamethasone, was used for expansion. H-CFU-C medium consisted of DMEM/F-12 supplemented with 1 × Insulin–Transferrin–Selenium, 10 mM nicotinamide, 2.5 mM HEPES buffer solution, 2 × penicillin streptomycin glutamine, and 0.1 mM non-essential amino acids. To induce the expansion of hepatic progenitor cell colonies, 0.25 μM A-83–01, 10 μM Y-27632, 40 ng/mL recombinant human HGF, and 20 ng/mL recombinant human EGF were added to induce the expansion of hepatic progenitor cell colonies. The medium was replaced every 3 days. After several expansions, expanded cells were used as human iPSC-derived hepatoblasts.

For the maturation of human iPSC-derived hepatoblasts, we used 3A and 3B mediums in the Cellartis iPS Cell to Hepatocyte Differentiation System (Takara Bio Inc.). According to the manufacturer's protocol, mixed 3A and 3B medium (3AB medium) was added to confluent hepatoblasts and cultured for 4 d. After incubation, the culture medium was replaced with Cellartis Hepatocyte Maintenance Medium (Takara Bio Inc.) and subsequently cultured for approximately 10 d.

### Promoter assay using luciferase expression vectors

The –1942, –1096, –583, and –281/ + 37 bp fragments from the transcription start site of the human tyrosine aminotransferase (*TAT*) promoter were amplified by PCR and cloned into the luciferase reporter vector, pGL4.10 (Promega, Madison, WI, USA).

As described previously^26^, HepG2 cells were cultured in DMEM containing 10% FBS and 1 × penicillin/streptomycin/glutamine (Invitrogen). The cells were seeded in 24-well tissue culture plates, grown to 90–95% confluency, and transfected with pGL4.10 reporter plasmid and pCAG-human KLF15 expression vectors using X-tremeGENE HP (Roche Diagnostics). As an internal control, the plasmid pRL-TK containing the Renilla luciferase gene was co-transfected. Cells were cultured for 48 h and then lysed with a passive lysis buffer (Promega). Luciferase activity was measured using the Dual-Luciferase Reporter Assay System (Promega) according to the manufacturer’s instructions.

### Immunocytochemistry

Cultured cells were washed with phosphate-buffered saline (PBS) and fixed with 4% paraformaldehyde in PBS. After three washes with PBS, cells were permeabilized with 0.25% Triton X-100 (Sigma)/PBS for 10 min, washed with PBS, and incubated with 5% donkey serum (Millipore, Bedford, MA, USA) in PBS for 1 h at room temperature. The cells were then incubated with diluted primary antibodies overnight at 4 °C. After washing with PBS, the cells were incubated with diluted secondary antibodies for 40 min at room temperature. Then, the cells were washed with PBS, and their nuclei were stained with 4’,6-diamidino-2-phenylindole dihydrochloride (DAPI; Sigma). The antibodies used for immunocytochemistry are shown in Supplementary Table 1. Colonies were imaged under a Carl Zeiss Axio Observer Z1 using AxioVision version 4.8 software (Carl Zeiss, Jena, Germany).

### Statistical analyses and guidelines

Statistically significant differences between samples were calculated using Student’s two-tailed t-test. Data are expressed as the mean expression ± standard deviation (SD). Statistical significance was set at P < 0.05 and < 0.01. All statistical analyses were performed using Microsoft Excel 2013 software and GraphPad Prism 7.04.

This study is reported in accordance with ARRIVE guidelines.

## Supplementary Information

Supplementary Information.
