# Supplementary material for: Kruppel-like factor 15 induces the development of mature hepatocyte-like cells from hepatoblasts
Source: Sci Rep. 2021 Sep 17;11:18551. doi: 10.1038/s41598-021-97937-6 (PMC8448749; doi:10.1038/s41598-021-97937-6)
Supplement: Supplementary file 1 — Supplementary Information. [file 41598_2021_97937_MOESM1_ESM.pdf]

Kruppel-like factor 15 induces the development of mature hepatocyte-like cells from hepatoblasts.

[a, b, #]Kazuya Anzai, [a, b, #]Kota Tsuruya, [a, c]Kinuyo Ida, [b]Tatehiro Kagawa, [c, d]Yutaka Inagaki, and [a, c]Akihide Kamiya*

[a]Department of Molecular Life Sciences, Tokai University School of Medicine, 143 Shimokasuya, Isehara, Kanagawa, Japan

[b]Division of Gastroenterology and Hepatology, Department of Internal Medicine, Tokai University School of Medicine, 143 Shimokasuya, Isehara, Kanagawa 259-1193, Japan.

[c]Center for Matrix Biology and Medicine, Graduate School of Medicine, Tokai University, 143 Shimokasuya, Isehara, Kanagawa 259-1193, Japan

[d]Department of Innovative Medical Science, Tokai University School of Medicine, 143 Shimokasuya, Isehara, Kanagawa 259-1193, Japan

Supplementary Tables 1 and 2

Supplementary Figures 1-7

Supplementary Table S1

List of antibodies used for experiments

**Primary antibodies for flow cytometry and MACS**	**Clone**	**Source**	**Catalog number**
biotin-conjugated anti-CD45	30-F11	Biolegend	103104
biotin-conjugated anti-Ter119	TER-119	Biolegend	116204
anti-Dlk1	24-11	MBL	D187-3<br>D187-4(FITC-conjugated)
CD133/1-APC	AC133	eBioscience	17-1331-81
Ter119-PE-Cy7	TER-119	eBioscience	25-5921-82
CD45-PE-Cy7	30-F11	eBioscience	25-0451-82
Kit-PE-Cy7	2B8	eBioscience	25-1171-82

**Primary antibodies for immunostaining**	**Dilution**	**Source**	**Catalog number**
α-fetoprotein (AFP)	1/300	Springer	E2950
ALB	1/400	Bethyl	A90-134A
HNF4α (C-19)	1/600	Santa Cruz	sc-6556
Sox9	1/200	Millipore	AB5535
Ki67	1/500	Abcam	ab15580

**Secondary antibodies**	**Dilution**	**Source**	**Catalog number**
anti-goat/Alexa Fluor 488	1/1000	Invitrogen	A11055
anti-rabbit/Alexa Fluor 555	1/1000	Invitrogen	A31572
anti-rabbit/Alexa Fluor 568	1/1000	Invitrogen	A10042

Supplementary Table S2

PCR primers for detection of mouse and human gene expression

**Mouse genes**	**Forward primer (5'→3')**	**Reverse primer (5'→3')**	Probe number
Hprt	tcctcctcagaccgctttt	cctggttcatcatcgctaatc	95
Tbp	ggcggtttggctaggttt	gggttatcttcacacaccatga	107
Klf15	acaggcgagaagcccttt	catctgagcgggaaaacct	64
Tat	ggaggaggtcgcttcctatt	gccactcgtcagaatgacatc	82
C/ebpα	aaacaacgcaacgtggaga	gcggtcattgtcactggtc	67
Hnf1α	cgcctccaccctggttat	actccccatgctgttgatg	98
Hnf4α	ccaagaggtccatggtgttt	ccgagggacgatgtagtcat	68
Foxa2	gagcagcaacatcaccacag	cgtaggccttgaggtccat	77
Mist1	ggctaaagctacgtgtccttg	ggtgaggcccttccaact	110
Klf5	ccggagacgatctgaaacac	cagatacttctccatttcacatcttg	17
Klf10	ggatgacagcttgcttccag	ggctgtaaggtggcgttaaa	95
Klf12	cctgccaaatgtgaccttaga	taccggggatggatgtacc	106
Cps1	gacaccactgcccgagac	cagcagacctgccacctt	95
Cyp3a11	gggactcgtaaacatgaacttttt	ccatgtcgaatttccataaacc	53
Cyp2b10	aagctcattctccagccaga	ctgtgggcaccaggaaag	106
Ck19	tgacctggagatgcagattg	cctcagggcagtaatttcctc	17
**Human genes**	**Forward primer (5'→3')**	**Reverse primer (5'→3')**	Probe number
TBP	cccatgactcccatgacc	tttacaaccaagattcactgtgg	51
TAT	ccatgatttccctgtccatt	ggatggggcatagccattat	37
CPS1	aatgtaatccgcctgctagtaaa	tccatcttggtgaaatcatgg	17
CYP1A2	ccagctgccctacttgga	gtgtcccttgttgtgctgtg	80
CYP2E1	caagccattttccacagga	caacaaaagaaacaactccatgc	67
KLF15	caaaagcagccacctcaag	tcagagcgcgagaacctc	64
P21cdkn1a	tcactgtcttgtacccttgtgc	ggcgtttggagtggtagaaa	32
P27cdkn1b	tttgacttgcatgaagagaagc	agctgtctctgaaagggacatt	60
P57cdkn1c	ctcctttccccttcttctcg	tccatcgtggatgtgctg	55
P14/16cdkn2a	gtggacctggctgaggag	ctttcaatcggggatgtctg	34
Tb53	gctcaagactggcgctaaaa	gtcaccgtcgtggaaagc	32
ALB	aatgttgccaagctgctga	cttcccttcatcccgaagtt	27
HNF4α	attgacaacctgttgcagga	cgttggttcccatatgttcc	3

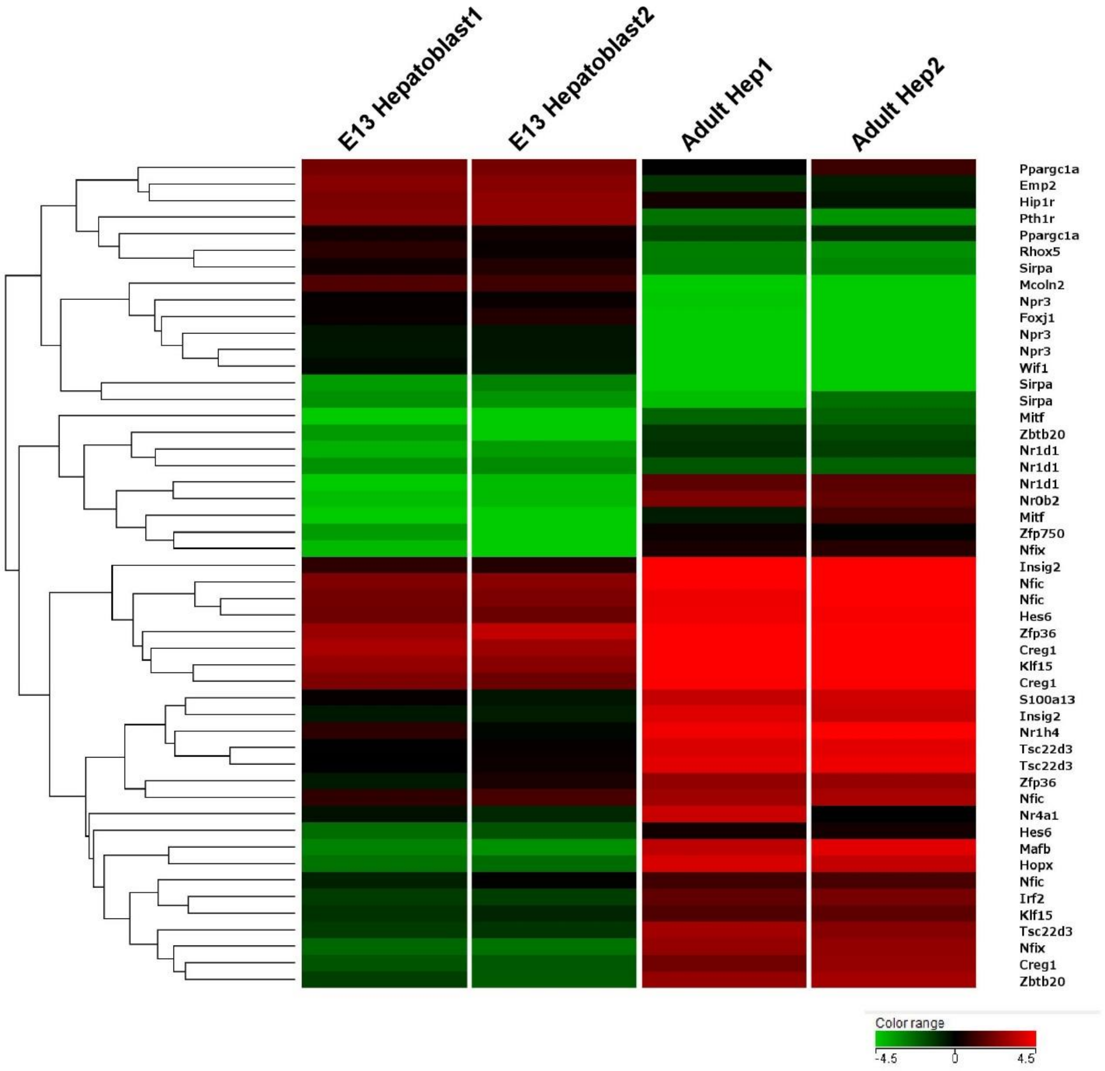

Sup Fig1. Kamiya et al.

Supplementary Figure 1 Transcriptional profiles of selected nuclear factors in mouse E13 fetal hepatoblasts and adult hepatocytes. Gene expression was analyzed by microarray analysis (n = 2). The probes of putative transcription factors are extracted and shown in the heat map.

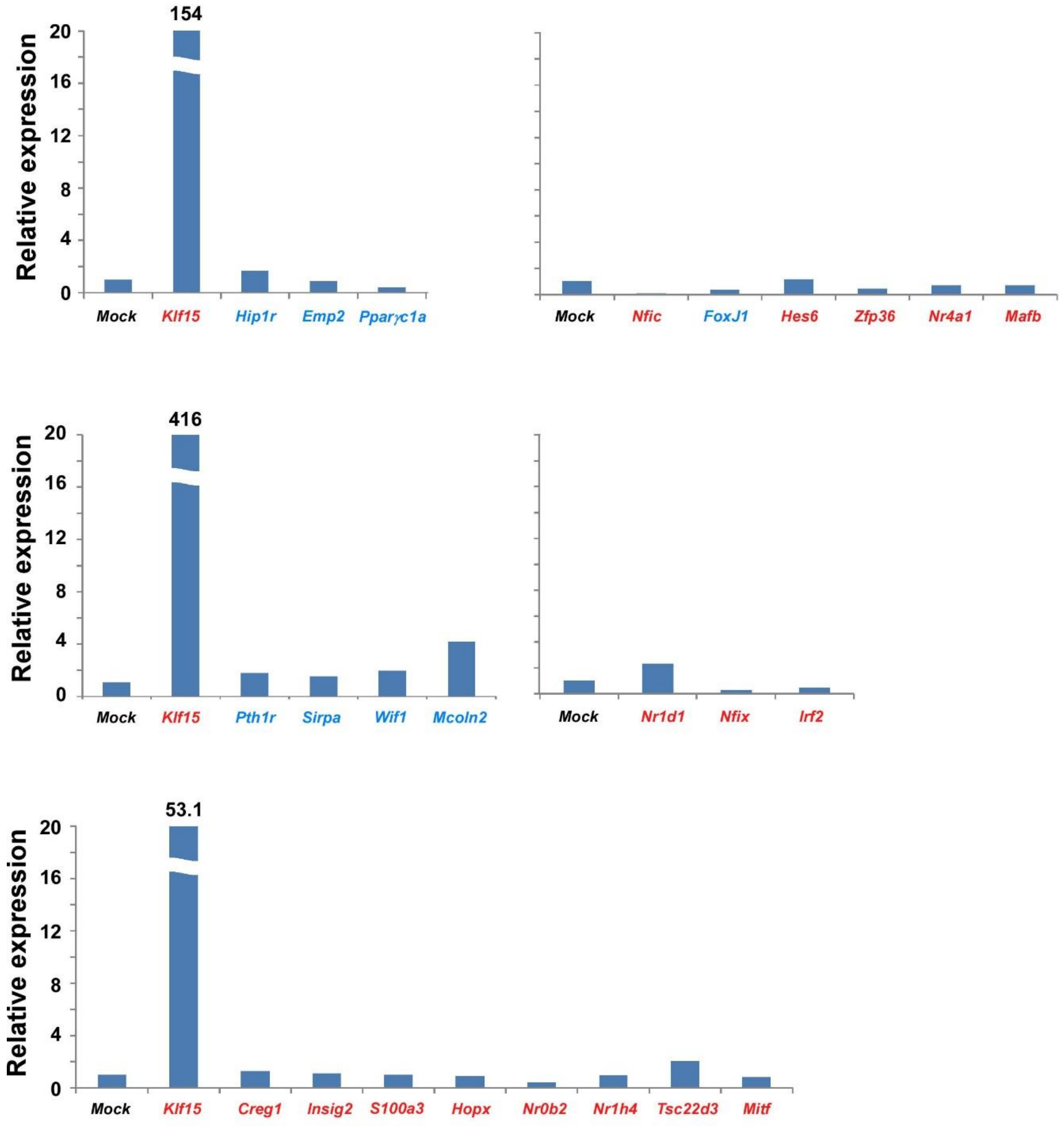

Sup Fig2. Kamiya et al.

Supplementary Figure 2 Maturation-inducing activity of the selected genes in mouse E13 fetal hepatoblasts culture. The individual gene was expressed in E13 hepatoblasts using retrovirus and expression of *Tat* was analyzed using quantitative RT-PCR after 7 days of culture. The expression of *Tat* in cells infected with the mock vector was set to 1.0.

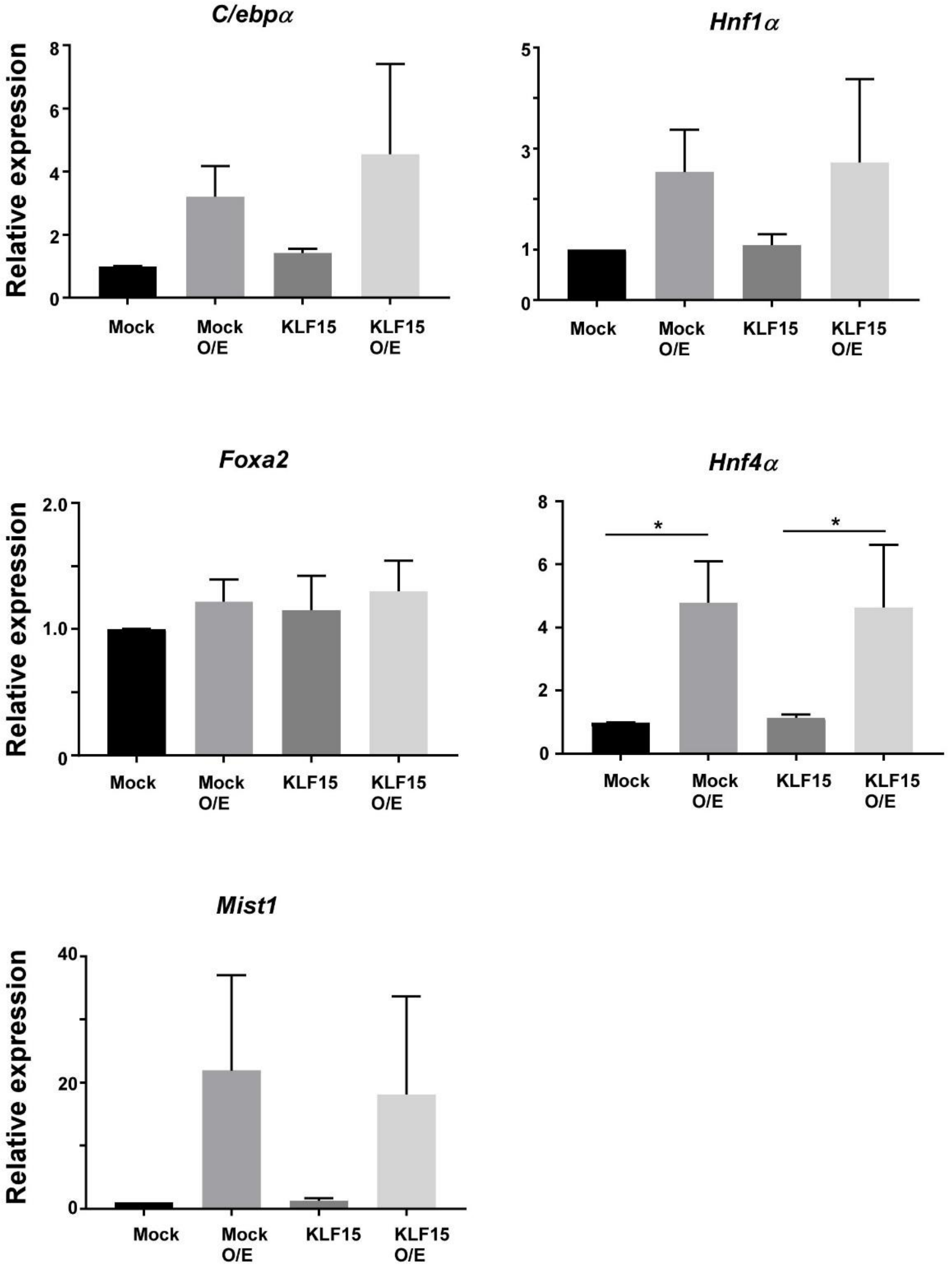

Sup Fig3. Kamiya et al.

Supplementary Figure 3 Expression of liver-enriched transcription factors in mouse primary hepatoblast culture with *Klf15*-overexpressing retroviral vectors. The expression of *C/EBPα, HNF1α, FoxA2, HNF4α,* and *Mist1* was analyzed using quantitative RT-PCR. Gene expression in cells infected with the mock vector was set to 1.0. Results are presented as the mean expression ± SD (n = 3). *P < 0.05.

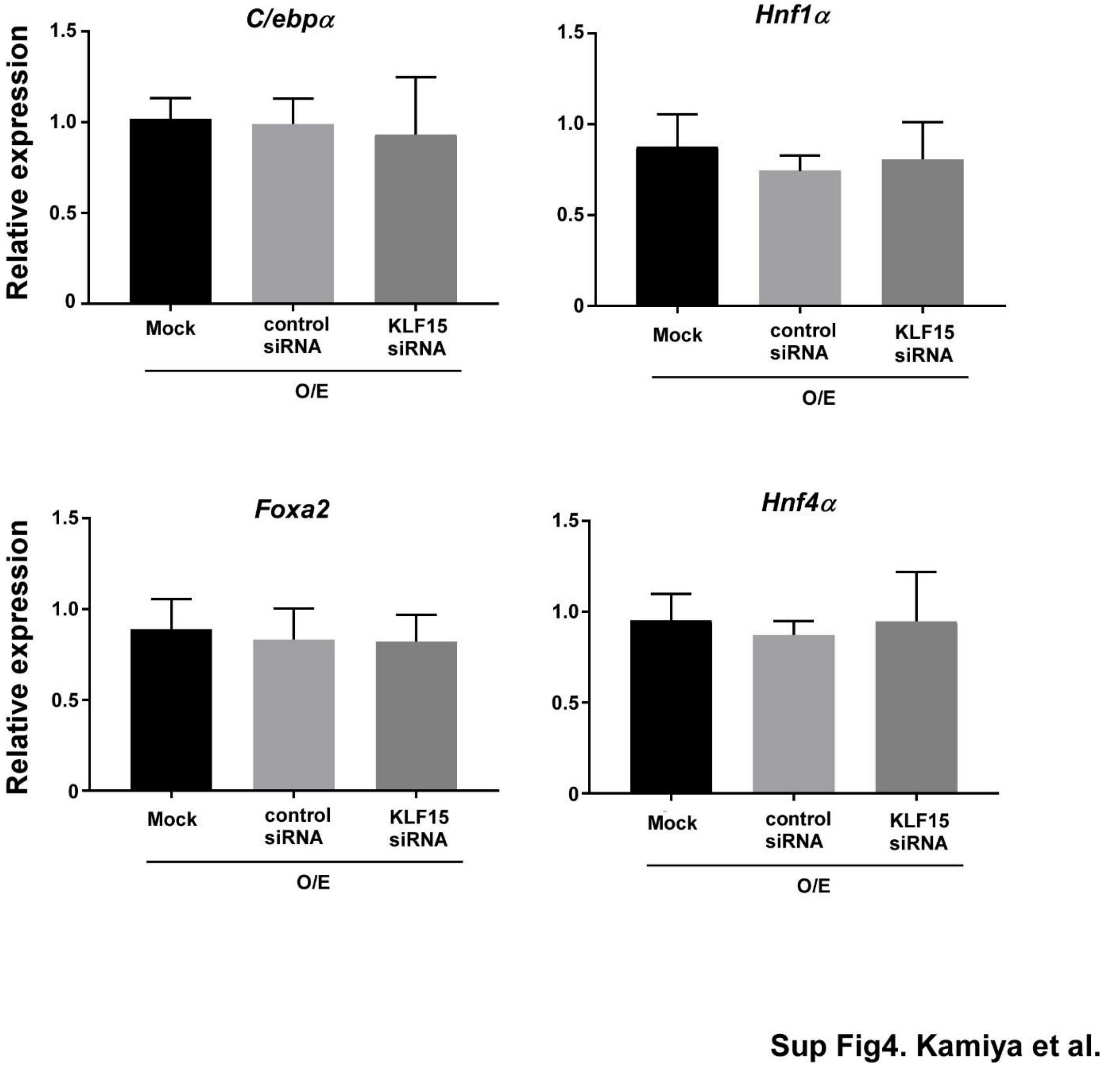

Supplementary Figure 4 Expression of liver-enriched transcription factors in hepatoblasts maturation culture with *Klf15* siRNA. Hepatoblasts were transfected with *Klf15* siRNA (same samples in Figure 2B), and the expression of *C/ebpα*, *Hnf1α, Foxa2*, and *Hnf4α* was analyzed using quantitative RT-PCR. Gene expression in cells infected with the control siRNA was set to 1.0. Results are presented as the mean expression ± SD (n = 3).

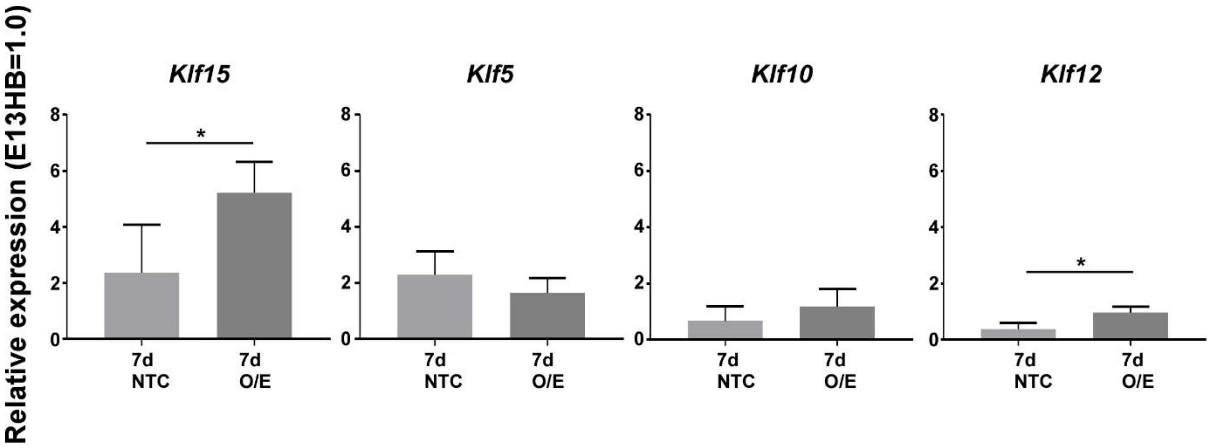

Sup Fig5. Kamiya et al.

Supplementary Figure 5 Expression of KLF transcription factors in E13 hepatoblast culture. The expression of *Klf15, Klf5, Klf10,* and *Klf12* was analyzed using quantitative RT-PCR. Gene expression in E13 primary hepatoblasts (n=2) was set to 1.0. Results are presented as the mean expression ± SD (n = 6). *P < 0.05.

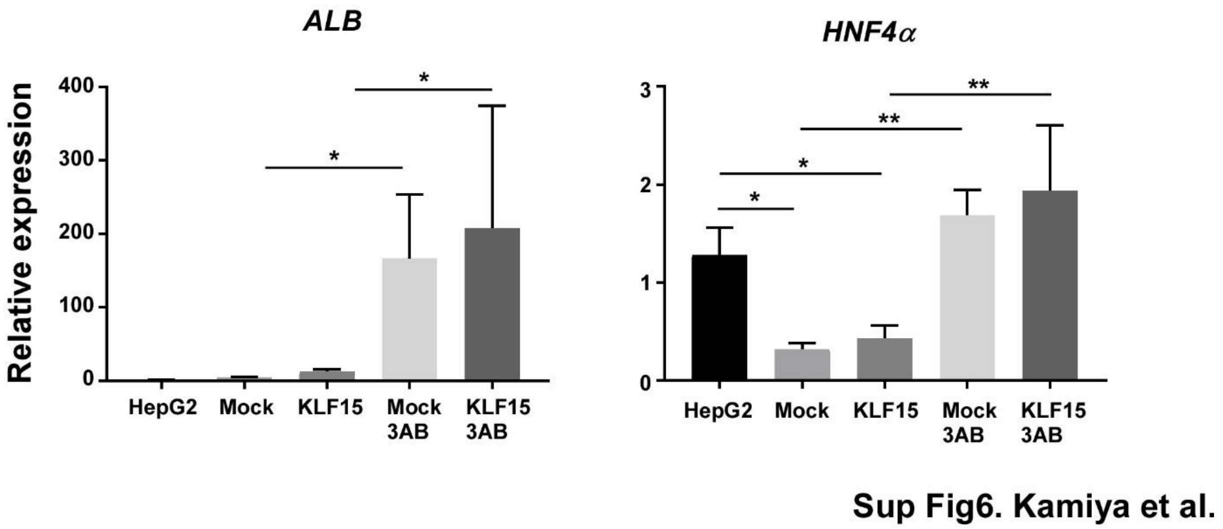

Supplementary Figure 6 Induction of hepatic marker genes in human iPSC-derived hepatoblasts culture. As shown in Figure 5A, after culturing, the expression of *ALB* and *HNF4α* was analyzed using quantitative RT-PCR. Gene expression in HepG2 cells was set to 1.0. Results are presented as the mean expression ± SD (n = 4; HepG2, n = 3). *P < 0.05, **P < 0.01.

A

```
GGCTGGAGTGCAATGGCATGATCACAGCTCTCTGCAGCCTTGACCTCCTGGGTTCAGGTGAT
CTTCCTACCTCAGCCTCCCAAGTAGCTGGGACTATAGGCTCATGCCACCATGCCTGGCTAGT
TTTTGTATCTTTTTGTATAGACGGGGTTTCACTATGTTGCCCAAGCTGGTCTTGAATTCCTT
AGCTCAAGTGATCCATCCACCTTGGCCTCCCGAAAGGCTGGGATTACCAAAGGTGTGAGCCA
CCATGCCCAGCCCCAAAATATTCTTTAAACATGTAATCATACAAAATTATATGAGATATTAA
CATTCTTTTTTTATACTAAGTCTTCAAAACCTGATGTGTATTTTACAACACATCTTAATTCA
GACCAGTCACATTTCAAGTATTCAACGGCCACTTATGACTAGTAGCTACAATATTAGACAGT
GCAACTATAGACCACGTTTACTCTCCATTTTTTTAGCAACTCCCAAACCAGCTTTATACGTA
TTGTAATAAACATAGAAAACATTTGGAAGGGTACACAAGAAATGACTAAAAATGATTTTTTT
ATATAAAATGAGAATGCAGGGAGGGAAGGTGATCAGGGTTTAATTCACGTACATCTCTATTT
GAATTTATTATATAAGGATATATCATATTTACTAAAAATGTAAAAGAGAAATGCTTATTCTT
TGCAACACAGTCCTTGTATAGAAATCTGTCTTAAGGAAATAATCAAATATGTAAACTAACAT
TTATGACAAGGATGCCCTTGGCCCAAGACGTGAATAATTTTTTCTAGTGTATCCATGCTATA
GACGTTACCTGCCATTACTGTATAAGAAAAAACATAGTGTACCTAGGGCTCAGTACTACCCA
TGGTTTCAGACATCCATAATTTCAGGCATCCACTGGAGGTCTTGGAGTGTGCTCCCTGTGGA
TAAGGGGGGACTACTGTATTCAACCCCACTGGAGGCTTGACTCCCTGAAAAGTGAATGGAGA
AGAACAAACTGATGTGAAAAATCTTTGTCTTCCAACAAATAAACAGACAGGATCTGAGCCCT
GGGGAGATCACTGAGGCTTCTCTTAACCCTTCAGTGGGTGGTATTTTCCATCTGCTCACAGG
AATGTTGAGATTTTGGCTGTGGACCTTGTGTTTTGCTTAGCAAAGTTGTTTTGTGTGGATCG
CACCTGACATTTTAAATTCTTCCAACAACCATAGGAGGTAAATGAAGAATTCATGCACAGTG
CAAGGATAAAACTGCACAGGTTTCAGCTGCCTTGGACACCCTGTGCTTTTCAGCTATTTGTT
GAATGGAACTTAAGTGCCCAATTTGTTCTGATTGGAGCAAAACTTTGTGGACTGACAAGGAT
GGGCGTCCAAGATGTAGGAAGTTAAAAAAAAAATGTTGCCAAACAAGGTGTAGAGCACGATC
CTATCTGTGTTTTGAAATCTATGAAATTATGTATCTATGCACCTATATGTAATGTATCTATC
TCTGAATGGAGGGAAAGAGCCTGACCATTACCACCCTCTACCCCTGGGAGTGAGAATGAAGT
GTACAGGGAGCAGGTGCAAGGAAGAATGCTCACTTCTTACTTTATATATTTCTGTACTGTTA
AAATTTTAACAGGCTGTTTTGTAAAAAAAATTTAATAAAAGCTATCAACTGTAACCAGAAGT
ACTTTCTGACAGTAGATGTTCTCAAACCATCTCACCAAGTGCCCAGAAAGAGTTAACACAGG
AGAATTCCAGATGTTTGAAGTGAGGACAAATTCCTGCTTTGAGGGTGGGAGGTGGGCTTAGG
GATGAGAGGCAATGACGGGGGAGAGGAGGAAAGAAGCTGAGGGGAGAGCTGGCTGCTGAGTT
GTCATTCCAACCAATGGCATGAAAGTTTCAAGCCCAACGCCCATTTGTGGAGACTATTTCAG
GAGTTAGGATTTGCATCTGAGTTCATTGCCCCTGTAACCTGTCAAAGAAGAG
```

B

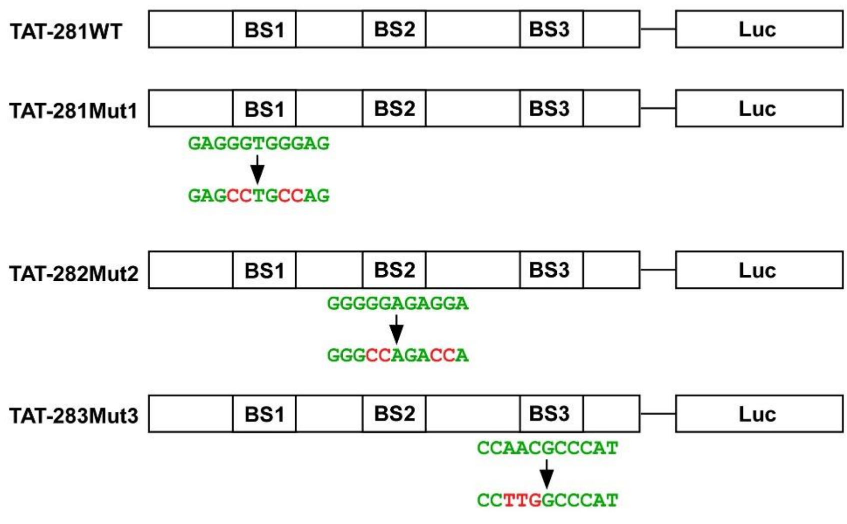

Sup Fig7. Kamiya et al.

Supplementary Figure 7 *KLF* consensus sequences in the promoter region of the human *TAT* gene. (A) Sequence of the -1943 bp upstream promoter region of *TAT*. The oligonucleotides used for vector construction of the truncated promoter region are shown in red. Predictive KLF binding sequences are shown as green oligonucleotides. The transcriptional start sequence is underlined. (B) Luciferase vectors with the wild-type (WT) and mutated -281 *TAT* promoter regions. Mut1, 2, and 3 vectors had KLF-binding consensus sequences (BS1-3), which had several oligonucleotide mutations as shown.
